# Could Climate Change Be Silently Damaging Medicines We Keep at Home?

**DOI:** 10.7759/cureus.116755

**Published:** 2026-09-22

**Authors:** Paulo Helito, Alanoud A Al-Baker, Zeyad Khalil, Taha Osman, Enzo Lorenti

**Affiliations:** 1 Radiology, Dar Al-Fouad Hospital, Cairo, EGY; 2 College of Medicine, Qatar University, Doha, QAT; 3 College of Medicine, October 6 University, Cairo, EGY; 4 Public Health, Ospedale “San Pio da Pietrelcina”, Villa d'Agri, ITA

**Keywords:** climate change, drug stability, extreme heat, household medicines, medication storage

## Abstract

Climate change is increasing the likelihood of medicines being exposed to temperatures outside recommended storage conditions after dispensing. Unlike controlled pharmaceutical storage and stability testing environments, everyday settings such as homes, vehicles, bags, delivery packages, domestic refrigerators, and periods of power interruption may subject medicines to fluctuating or extreme temperatures. The clinical significance of these exposures, however, remains uncertain and is likely to vary considerably between products.

This scoping review maps the available evidence on post-dispensing temperature exposure and medication stability, with particular attention to real-world storage conditions, physicochemical integrity, biological activity, potential clinical consequences, and practical strategies for reducing temperature-related risk. Evidence was considered across a range of temperature-sensitive and commonly used medicines, including insulin, refrigerated biologics, epinephrine, inhaled medicines, nitroglycerin, and oral formulations, together with studies evaluating household refrigeration, vehicle exposure, and low-resource cooling approaches.

The available literature indicates that medicines may frequently encounter conditions outside labelled storage recommendations after leaving formal supply chains. Nevertheless, exposure beyond a labelled temperature range should not be interpreted automatically as loss of pharmaceutical quality or clinical effectiveness. Thermal stability varies substantially according to formulation, temperature intensity, duration of exposure, cumulative thermal history, packaging, and storage conditions. Current evidence is strongest for selected temperature-sensitive medicines but remains fragmented across many other drug classes.

Overall, the literature supports a plausible climate-sensitive pathway linking increasing environmental heat with post-dispensing medication-quality risk, but evidence is currently insufficient to quantify the associated clinical burden at population level. Important priorities include characterising cumulative real-world thermal exposure, establishing clinically meaningful degradation thresholds, linking temperature histories with patient outcomes, and evaluating feasible adaptation measures. Climate-resilient medication management will require clearer storage guidance, improved patient education, practical cooling solutions, more reliable refrigeration, temperature-monitoring technologies, and stronger surveillance of medicines after dispensing.

## Introduction and background

Climate change is turning heat exposure from an episodic hazard into a persistent feature of daily life. The years 2015-2025 were the 11 warmest years on record, with 2025 approximately 1.43°C warmer than the 1850-1900 baseline [1], and the World Health Organization recognizes that heatwaves are becoming more frequent, prolonged, and geographically extensive [2]. Far less attention has been directed toward a related question: what happens to medicines themselves as the environments in which they are stored become hotter?

Importantly, the effects of elevated temperature and humidity are not uniform across pharmaceutical products. Even medicines containing the same active pharmaceutical ingredient may exhibit different stability profiles depending on their formulation and dosage form. Tablets, capsules, oral liquids, topical preparations, inhaled medicines, injectables, and biological products may respond differently to environmental stress. Liquid formulations may be particularly susceptible to processes such as hydrolysis, whereas solid dosage forms may show greater thermal resilience while remaining vulnerable to moisture-related degradation [3]. Biological medicines may be especially temperature-sensitive because their therapeutic activity can depend on maintaining complex three-dimensional molecular structures [4]. Consequently, the potential impact of climate-related heat exposure on medicines must be considered in relation to both the active ingredient and the formulation in which it is delivered.

Pharmaceutical stability is governed by temperature, humidity, light, formulation, packaging, and time. Elevated temperature can accelerate hydrolysis, oxidation, aggregation, and volatilization, with proteins carrying additional vulnerabilities such as conformational change. Regulatory stability testing establishes shelf life under specified conditions, but patient storage is far less controlled. A labelled storage condition is a boundary of demonstrated quality, not a guarantee of the environment a medicine will actually experience [5].

This gap is measurable. A Saudi Arabian study combining a survey of 1,005 households with temperature and humidity monitoring of 35 rooms across seven homes found that indoor temperatures exceeded 25°C for prolonged periods in several locations, while temperatures inside parked vehicles exceeded 70°C [6]. During a British heatwave, a drug cupboard reached average daily maximum temperatures of 30.7°C, whereas car boots reached mean daily maxima of 37.5-41.8°C, with individual peaks approaching 50°C [7]. Such conditions place medicines well beyond conventional “room temperature” storage environments and may accelerate temperature-dependent degradation processes. For many chemical degradation reactions, the relationship between temperature and degradation rate can be approximated using Arrhenius-type kinetics, whereby increasing temperature may substantially accelerate degradation [3]. However, this relationship is not universal across pharmaceutical products, and degradation behavior under fluctuating real-world conditions may be more complex than that observed under constant-temperature laboratory conditions.

Yet exceeding a labelled range does not mean a medicine instantly fails. Labels reflect submitted evidence, not a precise failure threshold - some products tolerate prolonged moderate excursions with little measurable change, while others are far more vulnerable, depending on duration, cycling versus constant heat, and formulation. Insulin provides a useful example: evidence suggests that some insulin preparations retain acceptable stability under moderate or fluctuating heat exposure, whereas prolonged constant heat can accelerate degradation; freezing remains a separate storage hazard [8-11].

This issue may be particularly important in settings where reliable electricity, refrigeration, or rapid medicine replacement is limited, because maintaining recommended storage conditions may be more difficult during periods of extreme heat [12]. This review maps evidence connecting household thermal exposure to medication stability after dispensing, distinguishing measured excursion from demonstrated degradation, and outlining adaptation and research priorities. Given the heterogeneity of the available evidence across medicine types, formulations, exposure conditions, stability outcomes, and study designs, a scoping review was undertaken to map the existing literature, identify evidence gaps, and clarify priorities for future research.

## Review

Review approach and scope

A structured scoping review approach was used because of the heterogeneity of the available evidence across medicine types, formulations, storage environments, exposure conditions, and outcomes. PubMed was used as the primary bibliographic database, and the final search update was conducted on 17 September 2026. The main search combined terms relating to medicine or pharmaceutical storage, temperature and environmental exposure, stability or degradation, and post-dispensing settings such as the home, patient transport, and vehicle storage. Because this broad environmental search did not retrieve all relevant product-specific stability studies, targeted supplementary PubMed searches were conducted for medicine groups central to the review, including insulin, epinephrine, inhaled bronchodilators, nitroglycerin, reconstituted oral antibiotics, and refrigerated biological medicines (Appendix 1). Reference lists of relevant reviews and included studies were also screened, and relevant WHO and ICH guidance was consulted.

Studies were considered eligible when they evaluated human medicinal products under post-dispensing, household, transport, vehicle, in-use, or experimentally simulated temperature conditions relevant to real-world storage, or directly assessed pharmaceutical stability, potency, physical integrity, or delivery performance following such exposure. Studies concerned solely with biological specimen storage, veterinary or food products, or formulation-development experiments without clear relevance to marketed medicines and real-world storage were excluded. Potentially relevant records were screened by title and subsequently assessed using abstracts or full texts where necessary. Evidence was organized into three tiers: environmental exposure, defined as documentation that a labelled storage threshold was exceeded; pharmaceutical effect, defined as a demonstrated change in potency, physicochemical stability, or delivery performance; and clinical effect, defined as a measurable patient outcome associated with the exposure. Evidence at one tier was not assumed to establish effects at the subsequent tier.

From ambient heat to the temperature a medicine actually experiences

Outdoor temperature is a poor proxy for what a stored medicine experiences. Alqurshi found Saudi household locations remaining above 25°C for prolonged periods and parked cars exceeding 70°C [6]. Crichton found similar amplification in a milder UK heatwave, with drug cupboards reaching 27.5-37.0°C and car boots up to 49.5°C [7]. The same applies in reverse - domestic refrigerators vary by shelf location and electricity reliability. Among 338 people with diabetes generating 400 temperature logs, most showed deviation from recommended ranges, and 17% recorded a reading below 0°C [8]; Santin et al. similarly found ~82.7% of rheumatology patients' home-stored biologics in inadequate conditions [13]. Not every setting shows major exposure, though: Dutch home-stored oral anticancer medicines mostly stayed within label conditions (Table 1) [14].

**Table 1:** Evidence that post-dispensing medicines experience non-standard thermal environments.

Study	Setting	Principal finding	Interpretation
Alqurshi (2020) [6]	Saudi households/vehicles	Indoor locations >25°C prolonged; parked cars >70°C	Major exposure potential in hot climates
Crichton (2004) [7]	UK heatwave	Cupboard maxima to 37°C; car maxima to 49.5°C	Microenvironments exceed ambient temperature
Vlieland et al. (2019) [14]	Netherlands	Anticancer medicines mostly within label	Risk varies by climate and infrastructure
Braune et al. (2019) [8]	US/EU insulin storage	78.8% of logs deviated; 17% <0°C	Cold exposure as relevant as heat
Santin et al. (2020) [13]	Brazil, home fridges (biologics)	82.7% inappropriate; -4.4°C to 15.5°C	Fridges cannot be assumed to hold 2-8°C

Insulin: the best-studied model of household thermal exposure

Insulin is one of the best-studied models of post-dispensing thermal exposure because many insulin products require refrigerated storage before use and are subsequently stored or carried by patients under variable environmental conditions. Manufacturers recommend protection from excessive heat and freezing, although labelled storage limits should not be interpreted as precise failure thresholds. The 2023 Cochrane review by Richter et al. found that several human insulin preparations retained acceptable activity under conditions above standard refrigeration, including oscillating temperatures of 25-37°C for up to three months; manufacturer data also indicated that some unopened short- and intermediate-acting human insulins could remain within acceptable potency limits for up to six months at 25°C [9]. These findings are product- and formulation-specific and should not be generalized to all insulin preparations.

The contrast between cyclic and constant heat exposure is particularly important. Kaufmann et al. reported that multiple insulin preparations exposed to a 25-37°C cycling profile retained approximately 96-102% potency after 12 weeks, whereas insulin maintained under constant elevated temperatures around 31°C showed approximately 5-19% potency loss over four to eight weeks [10]. Similarly, Vimalavathini and Gitanjali observed approximately 14-18% loss of regular human insulin after 28 days at 32-37°C [11]. These findings indicate that peak or mean temperature alone may not adequately characterize thermal stability, as the duration and pattern of exposure may also influence degradation. However, the comparison should be interpreted cautiously because the cyclic and constant-temperature experiments differed in exposure duration and other experimental conditions; therefore, the available evidence does not establish that temperature cycling is intrinsically protective.

Kongmalai et al.'s randomized crossover trial found insulin glargine stored at 37°C for 21 days was clinically equivalent to refrigerated insulin on continuous glucose monitoring [15], though specific to one product. Over 12 months, Christopher et al. found both regular human insulin and glargine degraded substantially at a constant 42°C, though glargine retained more activity, confirming thermostability is formulation-specific [16]. Insulin pump reservoirs run ~25-37°C, averaging ~30°C, from body heat [17]; heat plus agitation can cause visible agglomeration in suspensions [18]. Degradation is cumulative - shelf life shortens progressively as temperature rises [19].

Where refrigeration is unreliable, low-cost evaporative cooling may provide a practical alternative for reducing insulin storage temperatures. In a small preliminary study, outdoor temperatures of 34-43°C were reduced to approximately 25-27°C inside an unglazed clay pot, and insulin stored under these conditions produced glucose-lowering effects comparable to refrigerated insulin in eight healthy volunteers. In a separate real-world study involving six families in India, clay-pot storage reduced mean temperatures by approximately 2.6°C compared with open-box storage and was associated with a smaller decline in relative insulin potency after four months (0.5% versus 3.6%). Other evaporative systems have also shown cooling potential; Taerahkun and Sriphrapradang reported that a double-clay-pot device filled with wet sand achieved a relative cooling effect of 63.6%, compared with 57.7% for a commercial cooling wallet [20,21-23].

The central lesson: heat exposure should still be avoided, but consequences depend on formulation, duration, and exposure pattern - not the thermometer reading alone (Table 2).

**Table 2:** Selected evidence on insulin stability under non-standard storage conditions.

Study	Exposure/duration	Main findings
Richter et al. (2023) [9]	25-37°C, days-months	Many preparations retained potency longer/hotter than labels imply
Kaufmann et al. (2021) [10]	Oscillating 25-37°C for 12 weeks; constant elevated-temperature conditions for shorter periods	Insulin preparations retained potency under the oscillating profile, whereas some preparations showed measurable potency loss under sustained constant heat
Vimalavathini and Gitanjali (2009) [11]	5-37°C, 28 days	Deterioration at 32-37°C
Kongmalai et al. (2022) [15]	Constant 37°C, 21 days	Efficacy equivalent to refrigerated insulin
Christopher et al. (2024) [16]	4/22/42°C, 12 months	Progressive degradation; glargine more stable at 42°C
Herr et al. (2014) [17]	Normal pump wear, 35 days	Reservoirs averaged ~30°C
Al Shaibi et al. (1999) [20]	Clay-pot ~25-27°C, 6 weeks	Activity comparable to refrigerated insulin
Pendsey et al. (2023) [23]	Fridge/open box/clay pot, 4 months	Clay pots limited loss to ~0.5% vs. 3.6%
Braune et al. (2019) [8]	Household refrigeration/carriage	78.8% of logs deviated; 17% <0°C
Chandler et al. (2008) [24]	Simulated transit, 24-120 h	Within USP potency limits

Epinephrine: stability matters most when treatment cannot fail

Epinephrine auto-injectors are carried continuously so that an effective dose is available during a rare but potentially fatal emergency. Parish et al.’s systematic review found that epinephrine degradation under heat exposure depends on both temperature and duration, with prolonged constant heat producing greater degradation [25]. Rachid et al. found that auto-injectors stored at 70°C for 5 to 10 days delivered reduced epinephrine doses [26], a finding relevant to extreme storage environments such as parked vehicles. Because adequate epinephrine delivery is critical during anaphylaxis, heat-related changes in auto-injector performance may have important clinical implications.

Inhalers and drug-delivery devices

Metered-dose inhalers demonstrate that climate-related pharmaceutical risk can involve the delivery system as well as the active drug. Hoye et al. found that six months of storage in a vehicle exposed to extreme environmental temperatures produced a modest increase in propellant leakage but did not significantly alter emitted particle size, dose per actuation, or respirable mass when the inhalers were subsequently tested at room temperature [27]. In contrast, when the inhalers were actuated at temperatures outside the recommended range, including 47°C and 60°C, particle size, dose per actuation, shot weight, and aerosol mass distribution changed substantially. More directly, Ammari et al. evaluated salbutamol inhalers used and stored by patients under real-world conditions and found that summer exposures ranging up to 40.4°C were associated with a 12.3% reduction in delivered dose, together with reduced fine-particle dose and altered aerodynamic particle-size distribution [28]. These findings indicate that temperature may affect inhaler performance through both formulation and device-related mechanisms, potentially altering aerosol characteristics and the dose available for lung deposition. However, direct evidence linking such changes to clinical asthma outcomes remains limited.

Nitroglycerin: the importance of packaging

Sublingual nitroglycerin is vulnerable to volatilization and container interactions. Nawarskas et al. found faster potency loss with high-temperature storage and pocket-carrying [29]. Le et al. evaluated a purpose-built light-proof, airtight, inert container in which tablets remained stable across -20°C to 60°C for a week [30] - showing container-closure integrity can substantially offset environmental vulnerability.

Common oral medicines: a neglected household problem

Evidence on the stability of common oral medicines under realistic household storage conditions remains limited. Archibald and Brown stored ibuprofen tablets and suspensions in a household bathroom for a year and found progressive potency loss - to ~73% for tablets and ~83% for suspensions after 12 months - without detecting a toxic degradation product [31]. This shows degradation may simply mean less active ingredient - a subtle concern for wide-therapeutic-window drugs but greater for narrow-therapeutic-index medicines.

Liquid antibiotics and temperature-dependent instability

Reconstituted oral medicines deserve particular attention because their stability can become strongly dependent on storage conditions once water is added. Mack et al. found that commercially available amoxicillin-clavulanate suspensions remained substantially more stable under refrigeration than at warmer ambient temperatures. After seven days, amoxicillin degradation averaged approximately 1.4% at 8°C compared with 15.3% at 28°C, while clavulanic acid showed substantially greater temperature-related degradation [32]. These findings demonstrate how inappropriate household storage can compromise reconstituted oral formulations during a normal treatment course. Sub-potent antibiotic exposure raises a theoretical concern regarding antimicrobial resistance, although direct evidence linking climate-related pharmaceutical degradation to resistance remains insufficient.

Refrigerated biological medicines: heat and freezing are dual threats

Self-administered biological medicines have transferred part of the cold chain into patients’ homes, where maintaining recommended refrigerator temperatures can be difficult. Santin et al. found that 82.7% of patients did not maintain their biological medicines under adequate home-storage conditions, with recorded temperatures ranging from -4.4°C to 15.5°C [13]. Similarly, de Jong et al. found that only 11.6% of 276 golimumab injectors remained continuously within the recommended temperature range during transport and home storage [33]. Temperature excursions may have consequences that are not apparent from drug concentration alone: Vlieland et al. reported increased submicron or micron-sized particle numbers in 10 of 21 TNF-α inhibitor samples exposed to freezing or repeated freeze-thaw stress, despite some conventional analytical methods showing no differences from controls [34]. These findings highlight both inappropriate real-world storage and the potential for physical instability of biological medicines after temperature excursions.

Temperature excursion is not synonymous with drug failure

A central methodological conclusion of this review: label excursion, pharmaceutical degradation, and clinical failure are different outcomes. A logger reading 32°C beside a medicine labelled for storage below 25°C proves the label was exceeded, nothing more. This explains apparently contradictory findings: insulin stays acceptable after weeks of oscillating tropical exposure [9,10] yet degrades under constant heat [10,11]; epinephrine tolerates some excursions but fails under severe prolonged heat [25,26]; and nitroglycerin depends heavily on packaging [29,30].

Why climate change could amplify pharmaceutical instability

The proposed pathway is that climate warming may increase indoor and transport temperatures and thereby increase the likelihood of medicines experiencing storage conditions outside their labelled ranges. Depending on the product, formulation, duration, and pattern of exposure, such excursions may contribute to pharmaceutical degradation or altered delivery performance, with potential clinical consequences at the end of the pathway. Evidence is strongest for the environmental-exposure and pharmaceutical-stability links and remains much weaker for direct clinical outcomes. Heatwaves may also increase electricity demand and coincide with power disruptions that compromise refrigeration; WHO has recognized such risks in its 2026 heatwave assessment [35]. Climate change is therefore best understood as a risk multiplier rather than a novel mechanism of pharmaceutical degradation.

Discussion

This review identifies a credible but incompletely quantified threat to pharmaceutical quality after dispensing. Medicines clearly encounter temperatures well outside labelled conditions in homes, refrigerators, vehicles, and worn devices [6-8,14,17], and heat can affect potency or delivery performance, though the magnitude varies by formulation. Insulin's evidence base is strongest: many formulations appear more resilient than labels imply under moderate fluctuating heat [9,10,15,23], while sustained high heat still causes measurable degradation [10,11,16]. Evidence for epinephrine, inhalers, nitroglycerin, oral medicines, and biological products is more limited, but collectively demonstrates that adverse storage conditions can produce product-specific changes in potency, physical stability, or delivery performance [13,25-34]. Recent real-world evidence from Qatar further illustrates that environmental exposure does not uniformly translate into pharmaceutical failure. Makhlouf et al. evaluated more than 1,600 samples of 13 critical-care medicines stored for one year in operational rapid-response vehicles exposed to heat, humidity, vibration, and light. Ten medicines remained within compendial limits, whereas epinephrine, furosemide, and insulin showed the greatest variability, reinforcing the product-specific nature of environmental stability [36].

Pharmaceutical research asks how stable a product is at a given temperature; climate-health research asks how hot environments are becoming. Closing that gap requires continuous logging of the actual thermal history of medicines inside kitchens, bags, vehicles, and refrigerators, since a medicine cycling between 22°C at night and 38°C by day differs meaningfully from one held continuously at 30°C. Maximum temperature, duration above threshold, cycling frequency, and freezing events should all be recorded, alongside a summary index such as mean kinetic temperature.

The most important limitation is a causal evidence gap between environmental exposure, pharmaceutical degradation, and clinical outcome. Current evidence largely consists of three separate streams: environmental studies showing that medicines can experience temperatures beyond labelled storage conditions; laboratory studies demonstrating that specific medicines may undergo chemical degradation or performance changes under elevated temperatures; and a much smaller body of clinical evidence linking a documented temperature excursion in an individual patient’s medicine to confirmed degradation of that same product and subsequently to a measurable clinical consequence. Evidence from the first two streams does not, by itself, establish the third. For example, hyperglycaemia during a heatwave may result from dehydration or other physiological effects of heat rather than degraded insulin, while an asthma exacerbation may reflect ozone, aeroallergens, or heat-related respiratory stress rather than impaired inhaler performance. The current literature therefore supports concern, surveillance, and preparedness, but does not establish a widespread clinical burden directly attributable to heat-damaged medicines.

A heat-focused review may also overlook other environmental stresses relevant to pharmaceutical stability. Humidity is an established stability variable, particularly for moisture-sensitive products, and light exposure may also affect susceptible medicines [5]. Cold exposure remains important because medicines stored in domestic refrigerators may experience temperatures below their recommended range, including freezing events documented during home insulin storage [8]. A climate-resilient approach to medication storage should therefore consider multiple environmental exposures rather than heat alone.

Storage instructions such as “store below 25°C” are scientifically and regulatorily precise because they define the validated storage condition, but they do not necessarily provide patients with a practical decision pathway for every possible temperature excursion. Questions such as whether a medicine remains usable after two hours at 34°C or following a temporary refrigerator failure may therefore remain unanswered at the point of use. This issue may become increasingly important as climate change makes temperature excursions that were previously uncommon more frequent. Because product-specific excursion data are often not readily accessible to patients, usable medicines may be discarded unnecessarily, whereas potentially compromised products may be retained because they appear unchanged. Where supported by appropriate stability data, standardized and accessible excursion-management guidance derived from manufacturer-held evidence could help inform safer decisions after temporary deviations from labelled storage conditions.

Visible changes can indicate pharmaceutical deterioration, but their absence does not establish preserved potency. In a thermal-degradation study of injectable epinephrine, cyclic heat exposure produced a 31% reduction in epinephrine concentration without visible discoloration, demonstrating that substantial chemical degradation may occur while a medicine still appears normal [37]. Visual inspection should therefore not be used alone to determine whether a medicine remains suitable after significant temperature exposure.

Improved storage literacy is the most immediate step - patients should know that bathrooms, sunlit surfaces, and parked vehicles are inappropriate storage sites for most medicines, and refrigerated products should avoid placement against cooling elements. Passive evaporative cooling, such as clay pots, can substantially reduce temperatures at very low cost where electricity is unreliable [20-23]. Time-temperature indicators, proposed by Alqurshi for hot-climate households [6], can provide objective evidence of cumulative thermal burden, and packaging innovation may offer a more scalable intervention than universal refrigeration (Table 3).

**Table 3:** Climate-resilient strategies for household medication storage.

Intervention	Problem addressed	Advantages	Limitations
Product-specific excursion guidance	Uncertainty after heat exposure	Reduces unnecessary disposal	Needs manufacturer data
Patient education on storage location	Kitchens, bathrooms, vehicles	Low cost, immediate	Can't fix structurally hot homes
Domestic refrigerator education	Freezing and warm zones	Prevents placement errors	Fridge performance still variable
Passive clay-pot/evaporative cooling	No reliable refrigeration	Cheap, low-energy	Humidity-dependent
Validated insulated travel containers	Transport exposure	Portable, practical	Ice packs may freeze products
Time-temperature indicators/loggers	Unrecognized cumulative exposure	Objective thermal history	Validation and cost barriers
Climate-resilient packaging	Heat, moisture, light	Passive supply-chain protection	Needs engineering validation
Heatwave medication-storage advice	Short extreme-risk periods	Fits public heat alerts	Needs coordinated messaging

Expanding refrigeration indiscriminately could raise energy use and emissions, while overly conservative disposal after minor excursions increases waste. Better characterization of true thermostability - as with insulin - could simultaneously improve access and sustainability, since revised guidance based on genuine product-specific data could reduce refrigeration dependence where evidence supports it.

Future research should move beyond isolated stability-chamber experiments toward integrated exposure science that links the real storage environment, actual product exposure, pharmaceutical response, and potential clinical relevance. Calibrated data loggers should be deployed alongside medicines across different climates and electricity-reliability settings, because ambient weather-station measurements may not reflect the conditions experienced by medicines stored inside homes, bags, cupboards, refrigerators, or vehicles. Exposure monitoring should include relative humidity and, where relevant, light intensity in addition to temperature, and experimental protocols should reproduce realistic fluctuating environmental profiles rather than relying solely on fixed constant-temperature conditions. Newer self-administered products, including concentrated insulins, GLP-1 receptor agonists, and monoclonal antibodies, also warrant dedicated stability research because their formulations, devices, and storage requirements may differ from those of older insulin preparations. Study endpoints should extend beyond drug concentration to include aggregation, sterility, physical integrity, and device performance where applicable.

The literature's strength is convergence across environmental, pharmaceutical, and limited clinical evidence, particularly for insulin. Its weakness is heterogeneity - differing temperatures, durations, assays, and settings - along with heavy reliance on manufacturer data not always available at useful granularity. Evidence from hot, low-resource environments is particularly limited despite likely greater exposure. The literature supports risk mapping more than burden estimation.

Climate-related pharmaceutical stability can be integrated into heat-health preparedness through four dimensions: medicine susceptibility (proteins and suspensions differ from stable tablets), exposure intensity (actual microenvironment temperature, not outdoor weather), exposure duration (degradation is kinetic and cumulative), clinical criticality (the clinical consequences of reduced potency may be greater for medicines used in time-critical or life-threatening situations, such as emergency epinephrine). These explain why risk concentrates in temperature-sensitive essential medicines, prolonged heat, vulnerable housing, and unreliable electricity. WHO's reassessment of insulin thermostability offers a model for revisiting other essential medicines' storage requirements in hot-climate settings [12].

At the policy level, heat-health action plans could add a medication-storage component alongside existing hydration and cooling guidance - reminding patients not to leave medicines in vehicles and to seek pharmacist advice after significant excursions rather than automatically discarding or continuing a questionable product. Pharmacists are well placed to translate stability science into practical guidance at dispensing.

The question posed in the title cannot yet be answered with a universal yes or no: climate change is increasing the probability of household conditions capable of damaging some medicines, and such damage has been demonstrated experimentally, but how often it produces clinically important failure in real patients remains largely unknown - a surveillance gap rather than a reassurance.

## Conclusions

Climate change may increase the likelihood that medicines experience storage conditions outside labelled ranges after dispensing, but a temperature excursion does not itself establish pharmaceutical failure. Some insulin preparations retain acceptable stability under specified moderate or fluctuating heat exposures, whereas prolonged or more extreme heat can cause degradation; evidence for other medicines remains more limited and product-specific. Direct evidence linking climate-related storage excursions to clinically important patient outcomes remains sparse. Climate change is a risk multiplier, not proof that medicines are failing; better storage education, appropriate cooling strategies, and accessible product-specific excursion data are needed because post-dispensing storage remains a comparatively uncontrolled part of the medicine-use pathway.

## Appendices

Appendix 1. PubMed Search Strategy

Database: PubMed

Final search date: 17 September 2026

Search fields: Primarily Title/Abstract

Filters: No additional date, language, article-type, or species filters were applied.

Reported counts: Raw PubMed results at the time each search was conducted, before screening and removal of overlapping records.

A broad PubMed search was used to identify literature concerning post-dispensing medication storage and environmental temperature exposure. Because the broad search did not retrieve all relevant medicine-specific stability studies, targeted supplementary searches were conducted for insulin, epinephrine, inhaled bronchodilators, reconstituted oral antibiotics, nitroglycerin, and refrigerated biological medicines.

A1. Main post-dispensing medication-storage search

Search string:

(("drug storage"[Title/Abstract] OR "medication storage"[Title/Abstract] OR "medicine storage"[Title/Abstract] OR "pharmaceutical storage"[Title/Abstract] OR "storage conditions"[Title/Abstract] OR "temperature excursion"[Title/Abstract] OR "temperature excursions"[Title/Abstract]) AND (temperature[Title/Abstract] OR temperatures[Title/Abstract] OR heat[Title/Abstract] OR thermal[Title/Abstract] OR refrigeration[Title/Abstract] OR freezing[Title/Abstract] OR humidity[Title/Abstract]) AND (home[Title/Abstract] OR household[Title/Abstract] OR patient[Title/Abstract] OR patients[Title/Abstract] OR vehicle[Title/Abstract] OR vehicles[Title/Abstract] OR transport[Title/Abstract] OR "real-world"[Title/Abstract] OR "post-dispensing"[Title/Abstract]))

Results retrieved: 839

During title screening of the broad search, 239 records were retained for closer title-level assessment. Of these, 51 were considered clearly relevant, 29 potentially relevant, and 159 were excluded. The resulting 80 records progressed to abstract assessment; 52 were retained and 28 excluded at that stage. Medicine-specific supplementary searches were subsequently used to identify relevant studies that were not captured adequately by the broad environmental search.

A2. Targeted insulin search

Search string:

insulin[Title] AND (storage[Title/Abstract] OR stability[Title/Abstract] OR thermostability[Title/Abstract]) AND (temperature[Title/Abstract] OR heat[Title/Abstract] OR thermal[Title/Abstract] OR refrigeration[Title/Abstract] OR freezing[Title/Abstract])

Results retrieved: 343

A3. Targeted epinephrine search

Search string:

(epinephrine[Title/Abstract] OR adrenaline[Title/Abstract] OR "epinephrine auto-injector"[Title/Abstract] OR "epinephrine autoinjector"[Title/Abstract]) AND (storage[Title/Abstract] OR stability[Title/Abstract] OR degradation[Title/Abstract] OR potency[Title/Abstract] OR "dose delivery"[Title/Abstract]) AND (temperature[Title/Abstract] OR heat[Title/Abstract] OR thermal[Title/Abstract] OR freezing[Title/Abstract] OR cold[Title/Abstract])

Results retrieved: 191

A4. Targeted inhaler/bronchodilator search

Search string:

(inhaler[Title/Abstract] OR inhalers[Title/Abstract] OR "metered dose inhaler"[Title/Abstract] OR "metered-dose inhaler"[Title/Abstract] OR salbutamol[Title/Abstract] OR albuterol[Title/Abstract]) AND (storage[Title/Abstract] OR stability[Title/Abstract] OR performance[Title/Abstract] OR "dose delivery"[Title/Abstract] OR degradation[Title/Abstract]) AND (temperature[Title/Abstract] OR heat[Title/Abstract] OR thermal[Title/Abstract] OR humidity[Title/Abstract] OR freezing[Title/Abstract])

Results retrieved: 290

A5. Targeted reconstituted oral-antibiotic search

Search string:

("oral suspension"[Title/Abstract] OR "oral suspensions"[Title/Abstract] OR "reconstituted suspension"[Title/Abstract] OR "reconstituted suspensions"[Title/Abstract] OR amoxicillin[Title/Abstract] OR clavulanate[Title/Abstract] OR "amoxicillin-clavulanate"[Title/Abstract]) AND (stability[Title/Abstract] OR degradation[Title/Abstract] OR potency[Title/Abstract] OR storage[Title/Abstract]) AND (temperature[Title/Abstract] OR heat[Title/Abstract] OR refrigeration[Title/Abstract] OR refrigerated[Title/Abstract] OR "room temperature"[Title/Abstract])

Results retrieved: 225

A6. Targeted nitroglycerin search

Search string:

(nitroglycerin[Title/Abstract] OR "glyceryl trinitrate"[Title/Abstract]) AND (storage[Title/Abstract] OR stability[Title/Abstract] OR degradation[Title/Abstract] OR potency[Title/Abstract]) AND (temperature[Title/Abstract] OR heat[Title/Abstract] OR thermal[Title/Abstract] OR humidity[Title/Abstract] OR light[Title/Abstract] OR vehicle[Title/Abstract] OR pocket[Title/Abstract])

Results retrieved: 56

A7. Targeted refrigerated biological-medicines search

Search string:

(biologic[Title/Abstract] OR biologics[Title/Abstract] OR "biological medicine"[Title/Abstract] OR "biological medicines"[Title/Abstract] OR "monoclonal antibody"[Title/Abstract] OR "monoclonal antibodies"[Title/Abstract] OR "TNF-alpha inhibitor"[Title/Abstract] OR "TNF-alpha inhibitors"[Title/Abstract]) AND (storage[Title/Abstract] OR stability[Title/Abstract] OR "temperature excursion"[Title/Abstract] OR "temperature excursions"[Title/Abstract]) AND (temperature[Title/Abstract] OR heat[Title/Abstract] OR freezing[Title/Abstract] OR "freeze-thaw"[Title/Abstract] OR refrigeration[Title/Abstract])

Results retrieved: 1,079

A8. Supplementary identification of evidence

The targeted searches were supplementary to the main PubMed search and were intended to identify medicine-specific stability studies that could be missed by a broad post-dispensing environmental search. Consequently, overlap between the results of the main and targeted searches was expected, and the raw search counts should not be summed to estimate the number of unique records screened.

Reference lists of relevant reviews and included studies were also examined to identify additional eligible publications. Relevant World Health Organization and International Council for Harmonisation guidance documents were consulted where they provided information relating to pharmaceutical stability, storage conditions, temperature excursions, or heat-related risks.

Studies were considered for inclusion when they assessed human medicinal products under household, post-dispensing, patient-transport, vehicle, in-use, or experimentally simulated temperature conditions relevant to real-world storage, or when they directly evaluated pharmaceutical stability, potency, physical integrity, biological activity, or delivery performance following such exposure.
